# Transcriptional regulation of cancer stem cell: regulatory factors elucidation and cancer treatment strategies

**DOI:** 10.1186/s13046-024-03021-y

**Published:** 2024-04-02

**Authors:** Zhengyue Zhang, Yanjie Zhang

**Affiliations:** 1 Department of Oncology, Ninth People’s Hospital, Shanghai Jiao Tong University School of Medicine, Shanghai, 201900 People’s Republic of China; 2 Shanghai Institute of Precision Medicine, Ninth People’s Hospital, Shanghai Jiao Tong University School of Medicine, Shanghai, 200125 People’s Republic of China

**Keywords:** CSC, Transcription regulation, TF, Signaling pathways,epigenetics, DNA elements, TME

## Abstract

Cancer stem cells (CSCs) were first discovered in the 1990s, revealing the mysteries of cancer origin, migration, recurrence and drug-resistance from a new perspective. The expression of pluripotent genes and complex signal regulatory networks are significant features of CSC, also act as core factors to affect the characteristics of CSC. Transcription is a necessary link to regulate the phenotype and potential of CSC, involving chromatin environment, nucleosome occupancy, histone modification, transcription factor (TF) availability and cis-regulatory elements, which suffer from ambient pressure. Especially, the expression and activity of pluripotent TFs are deeply affected by both internal and external factors, which is the foundation of CSC transcriptional regulation in the current research framework. Growing evidence indicates that regulating epigenetic modifications to alter cancer stemness is effective, and some special promoters and enhancers can serve as targets to influence the properties of CSC. Clarifying the factors that regulate CSC transcription will assist us directly target key stem genes and TFs, or hinder CSC transcription through environmental and other related factors, in order to achieve the goal of inhibiting CSC and tumors. This paper comprehensively reviews the traditional aspects of transcriptional regulation, and explores the progress and insights of the impact on CSC transcription and status through tumor microenvironment (TME), hypoxia, metabolism and new meaningful regulatory factors in conjunction with the latest research. Finally, we present opinions on omnidirectional targeting CSCs transcription to eliminate CSCs and address tumor resistance.

## Background

CSC regulates cancer behavior to influence treatment outcomes and brings important and promising targets. The process of expressing stem genes in CSC involves transcription, post transcription, translation, and post translation stages, and the transcription process is of paramount importance and has an impact on other stages. Aiming to summarize the diverse links and targets of CSC transcription regulation, we mainly searched the PubMed database for literature related to CSC transcription over the past 5 years, and comprehensively discussed strategies and suggestions for targeting CSC transcription.

## Introduction

CSCs possess self-renewal and differentiation properties analogous to normal stem cells thus bringing high tumorigenic potency [1]. Tumors are considered to have hierarchical structure and the CSCs are located at the apex in CSC model [2]. CSCs occupy a very small amount in tumor tissue, and they have similar TFs and signaling pathways compared to stem cells in healthy tissues [3, 4]. One prominent hallmark of CSCs is asymmetric division, which generates more CSCs and specially-differentiated daughter cells [5, 6]. Another important feature of CSCs is epithelial-mesenchymal transition (EMT), a reversible cellular process that temporarily puts epithelial cells in a quasi-mesenchymal state. Conversely, the resulting mesenchymal cells can restore to an epithelial state, known as mesenchymal-epithelial transition (MET) [7]. New insights suggest that EMT can manifest as a mixed state of two processes, defined as epithelial mesenchymal plasticity (EMP), which enables CSC to achieve higher stemness [8]. After tumors have formed, CSCs tend to exist in a dormant state or G0 phase and be relatively static [4, 9]. CSCs have the capacity to transition between static and proliferative states [10]. It is shown that quiescent human glioblastoma (GBM) cancer stem cells are activated and accelerate the transition to a proliferative state after chemotherapy [11]. Besides, CSCs can not only secrete vascular endothelial growth factor (VEGF) or convert into vascular progenitor cells to induce angiogenesis, but also invade and infiltrate blood vessels and lymphatic vessels to achieve metastasis and dissemination [12]. In addition, owning to a comprehensive DNA repair system, enhanced DNA damage response (DDR) and decreased apoptosis, CSCs appear to be more resistant to the environment for therapy [13]. It is obvious that complicated tumor heterogeneity mainly originates from CSCs, and various malignant behaviors of tumors are integrally linked to CSCs, including tumorigenesis, progression, metastasis, drug resistance and recurrence [14].

The characteristics of CSC is closely related to the expression of pluripotent genes and the role of pluripotent TFs. Therefore, the understanding and regulation of CSC transcription is an important prerequisite for eliminating cancer stemness and treating cancer. Transcription in living cells is a highly dynamic and explosive process characterized by sporadic bursts interspersed with hours of inactivity [15].The transcriptional regulation of eukaryotes involves various molecules and mechanisms, including TFs (able to bind DNA, locate RNA enzymes, and recruit other regulatory factors), epigenetic modifications (alter the DNA and proteins of chromosomes through chemical modifications) [16, 17], genomic DNA elements (able to bind proteins) [18]. TF residence time and concentration have an impact on the frequency of bursts [19], which is considered as key controllable factor in transcription. The type and degree of epigenetic modifications have a significant impact on transcriptional activity, and diverse DNA elements are involved in the initiation and enhancement of transcription. In addition, RNA polymerase can regulate the size and efficiency of transcription bursts, responsible for stochastic fluctuations in protein levels [20], affecting the exertion of biological functions, but there is limited research in CSC. It is exposed that the microenvironment has bi-and unidirectional interactions with the plasticity of transcriptional heterogeneity, including hypoxia, metabolic alterations, and immune stress [21]. A study on GBM uncovers the spatial interdependence between tumors and hosts, confirming that environmental pressure can trigger adaptive transcription programs. This provides a theoretical basis for regulating CSC transcription through ambient pressure.

Targeting CSCs for treatment is a highly promising strategy to address the challenges of tumor resistance and recurrence. CSC can be inherited from stem cells in normal tissues or produced by mutations, and its state varies with the occurrence and development of tumors, while also having a decisive role in tumor behavior [22]. It is reasonable to alter the signaling and fate of CSC by clarifying its transcriptional regulation, thereby eliminating CSCs and preventing metastasis. With the proposal and development of spatiotemporal molecular medicine, further study on transcriptional regulation of CSC will assist us understand the development of drug resistance and seek new therapeutic drugs and methods [23]. Here, we will describe the transcriptional regulation characteristics of CSC from various aspects and how to target these stages to obtain therapeutic benefits.

### TFs and signaling pathways in the transcriptional regulation of CSC

CSC TFs can regulate the expression of stem genes by combining with the enhancer or promoter sequence on DNA, which is significant for the occurrence, invasion and metastasis of tumors [24]. A variety of signaling pathways in CSC can activate the expression of related TFs and facilitate the stability of TF protein to maintain the undifferentiated state and the nature of CSCs, or directly combine with DNA to promote the expression of stem genes and oncogene [4, 9]. Next, we will demonstrate the characteristics and effects of diverse TFs and signaling pathways regulating transcription in CSCs, and the schemes and benefits targeting them will be explored.

### Some embryonic TFs and signaling pathways are re-expressed or reactivated in CSC

The similarities between the processes of embryonic development and cancer have been discovered early. Research set about comparing the key signals and regulatory mechanisms in embryo and cancer, a lot of relevant and mature conclusions have been obtained. Figuring out the functions of these main TFs and signaling pathways is conducive to a better understanding of the characteristics and status of CSCs.

### Pluripotent TF

In embryonic stem cells (ESC), several pluripotent TFs are involved in normal organogenesis and control of cell fate, including octamer-binding transcription factor 4 (OCT4), sry-related HMG box 2 (SOX2), NANOG, kruppel-like factor 4 (KLF4) and MYC [25], which can reprogram somatic cells to become induce pluripotent stem cells by transient ectopic overexpression [4, 26]. Accumulating studies have revealed that pluripotent TFs are almost inhibited in adult tissues but overexpressed in invasive cancer, regulating the biological activity of CSCs and endowing them with many characteristics [25, 27]. Intervention against these TFs is a considerable approach for targeting CSC through transcriptional regulation.

OCT4, SOX2 and NANOG are considered as key regulatory factors to maintain the self-renewal, proliferation and differentiation of CSCs [28]. OCT4 is a member of Pit-Oct-Unc transcription factor family, encoded by POU5F1 gene, which is overexpressed in CSCs of various cancers [29]. A study of head and neck squamous cell carcinoma (HNSCC) reported that OCT4 was involved in the self-renewal of CSCs and DNA damage induced by irradiation, thus promoting the radiation resistance of tumor [30]. Experiments have revealed the transcriptional mechanism of OCT4 to maintain the stemness of GBM. The palmitoylation of OCT4A (a variant of OCT4) prevents it from lysosomal degradation and contributes to the integration of *SOX2* enhancer region, promoting the tumorigenicity of glioma stem cells (GSCs) [31]. In addition, OCT4 has been shown to induce CSCs to produce vascular endothelial cells and participate in the angiogenesis of hepatocellular carcinoma (HCC) [32]. A number of studies manifest that OCT4 is an indispensable stem TF in CSCs. SOX2 plays a crucial role in the early development of ESCs and cancer progression, belonging to the *SOX* gene family characterized by high mobility group (HMG) domain [33]. There is evidence that nicotine can induce the expression of *SOX2* to enhance the self-renewal of CSCs in non-small cell lung adenocarcinoma (NSCLC), and OCT4 may act on this process [34].By inhibiting the expression of *SOX2*, the characteristics of CSC can be repressed, making breast cancer cells sensitive to tamoxifen [35]. These studies demonstrate that SOX2 is a significant TF that mediates the proliferation and drug resistance of CSCs. NANOG is a differentiated homeobox (HOX) domain protein and one of the essential transcription factors in CSCs [36]. It is reported that NANOG is upregulated in sarcoma CSCs and promotes the formation of sarcoma cells, making it resistant to chemotherapy and radiotherapy, and this resistance can be overcome by inhibiting NANOG [37]. After the interaction of Abelson interaction factor 2 (ABI2) and TF MEOX2, MEOX4 and NANOG promoter regions bind to activate transcription, promoting the phenotype of CSCs and inducing tumor recurrence in HCC [38]. NANOG has been shown to be involved in tumor recurrence and treatment resistance. Moreover, the above three pluripotent TFs are beneficial for promoting EMT [39, 40], and correlate with the survival rate and prognosis of patients [41].

KLF4 and MYC are also cohesively involved in the transcriptional regulation of stem cells, development and cancer. KLF4 is a zinc-finger-containing TF, which contributes to embryonic development and stem cell generation and exists in a variety of human tissues [42]. KLF4 directly binds to the promoter to up-regulate the expression of *LAMA4*, which ultimately enhances the malignant behavior of osteosarcoma cells and inhibits apoptosis [43]. Knockdown of *KLF4* impairs the osteosarcoma CSCs and inhibits the osteosarcoma cells migration [44]. In contrast, *KLF4* was significantly down-regulated in oxaliplatin resistant colorectal cancer (CRC) cells [45]. The sensitivity of CRC cells to radiotherapy was enhanced and the stemness of CSCs was reduced after targeting *KLF4* [46]. KLF4 plays a role in different types of cancer as oncogenic or anticancer factor. MYC family proteins (C-MYC, N-MYC, L-MYC) are essential to maintain the properties of ESCs and CSCs [25], and the overexpression of *MYC* often leads to tumorigenesis. Research exposed that MYC combined with SLUG promoter activated the transcription, which increased the expression of *OCT4* and *NANOG*, and enhanced the characteristics of CSCs in breast cancer [47]. The combination of MYC-Interacting Zinc Finger Protein 1 (MIZ-1) and C-MYC can improve the efficacy of the pro-autophagy factor *AMBRA1* promoter, which will affect the stemness, growth and migration of CSCs in medulloblastoma (MB) [48]. MYC is unable to regulate transcription alone but works together with other genes and regulatory factors to exert effect on CSCs and cancer progression [49].

In addition, FOXM1, the only member of forkhead box protein M subfamily, has gradually accumulated abundant evidence for the regulation of CSCs. FOXM1 affects the temporal and spatial expression of genes during embryonic development while maintaining the homeostasis and repair in adult tissues [50]. FOXM1 has been reported to be dysregulated in a variety of CSCs [51]. In gastric cancer (GC) and nasopharyngeal carcinoma, FOXM1 induces the characteristics of CSCs and enhances the tumorigenicity [52, 53]. It is proved that *FOXM1* is down-regulated in CSCs of pancreatic cancer, thus making it sensitive to chemotherapy [54]. Growing studies indicate that FOXM1 acts as vital TF to regulate the properties of CSCs.

### Developmental signaling pathways

It has been identified that five common signaling pathways are expressed in ESCs and CSCs, which endow CSCs with the properties of proliferation and differentiation. They consist of Wnt/ β-Catenin, Notch, hedgehog (Hh), Hippo and TGF-β pathways, called oncofetal drivers [25, 55].

Wnt pathway is a key regulator of embryonic development and adult tissue differentiation, and also an effective driver of cancer [56]. The typical Wnt signal is transmitted to the nucleus through Frizzled (atypical G protein coupled receptor, GPCR) and LRP5/6 (LDL receptor related protein), which activates the intracellular protein Dishevelled (DVL) and inhibits the degradation activity of β-Catenin degradation complexes formed by serine-threonine protein kinase GSK3β [57]. With accumulation and translocation of β-Catenin, the expression of *MYC* and other stem genes is up-regulated to induce CSC proliferation [57, 58]. Atypical Wnt signal is transmitted to cells through Frizzled or ROR1/2 (receptor tyrosine kinase-like orphan receptor), which activates TFs of cyclins to induce CSC dormancy and fight apoptosis [57].

Notch signaling pathway is involved in growth and development, while acting as carcinogenic signal or tumor suppressor according to tumor type and stage [59, 60]. After the Notch ligand binds to the Notch receptor on the cell membrane, they undergo three cleavages and transforms into the Notch intracellular domain (NICD), which translocates to the nucleus and binds to the TF——CSL (CBF-1/suppressor of hairless/Lag1) [60]. CSL undertakes DNA binding and additional TFs recruitment to regulate gene expression. Five Notch ligands have been identified in humans, namely delta-like ligand 1 (DLL1), delta-like ligand 3 (DLL3), delta-like ligand 4 (DLL4), Jagged-1 (JAG1), and Jagged-2 (JAG2), which respectively mediate distinct cell activities [4, 60].

Hh pathway regulates the process of organogenesis and tumorigenesis, which is a complex signal network. There are three kinds of Hh family protein in mammals: Sonic hedgehog (Shh), Indian hedgehog (Ihh) and Desert hedgehog (Dhh). The components of typical Hh pathway include extracellular Hh ligand, twelve-span transmembrane protein Patched (PTCH1 and PTCH2), GPCR family transmembrane protein Smoothened (SMO), intermediate transduction molecules and Glioma-associated oncogene homolog (GLI) family of TFs (GLI1, GLI2 and GLI3) [4, 61]. In the presence of Hh ligand, the inhibition of PTCH on SMO is relieved, then SMO activates GLI to regulate the expression of target genes [61, 62]. GLI1 only acts as a transcriptional activator, enhancing the expression of SOX2 and NANOG to promote the formation of tumorsphere [63, 64]. GLI2 has dual functions but mainly activates transcription [4, 63], which can promote the stemness of renal cell carcinoma [65]. GLI3 is a transcription inhibitor that affects the growth and migration of cancer cells [66], and its knockdown reduces the stemness of oral squamous cell carcinoma [67].

Hippo pathway plays a major role in development and cancer [68]. Hippo involves kinase cascade reaction, and extracellular signal transduction affects MST1/2 (serine threonine kinase3/4) and LATS1/2 (large tumor suppressor kinase1/2) through various upstream activators or inhibitors [69]. After the dephosphorylation and nuclear translocation of YAP/TAZ (Yes-associated protein/trascriptional coactivator with PDZ-binding motif) are induced, various TFs, especially the transcriptional enhanced associate domain (TEAD) family, can be combined to guide gene expression (include OCT4 and NANOG) [70]. The decrease of YAP/TAZ level can down-regulate the expression of *SOX2* and other stem genes in small cell lung cancer (SCLC) CSCs, thereby enhancing chemosensitivity and cell death [71].

Transforming growth factor β (TGF-β) mediates embryogenesis and adult function, mainly uses SMAD protein as a receptor to regulate target genes with other signal proteins [72]. TGF-β can induce EMT and mediate the expression of phenotype proteins in CSCs, which promote the metastasis and progression of cancer [73, 74].

### Crosstalk of distinct TFs and signaling pathways regulates the transcription of CSCs

Multiple TFs are comprehensively activated to induce CSC function. After the interaction between palmitoylated OCT4a and SOX4, the activity of *SOX2* enhancer is increased to regulate the CSCs of GBM [31]. And the overexpression of *SOX2* increases the expression of key pluripotent factors OCT4 and NANOG [75]. It is exposed that the phase separation of NANOG and TAZ promotes the transcription of *SOX2* and *OCT4* to maintain CSCs stemness [76]. Besides, MYC can contribute to the up-regulation of *OCT4* and *NANOG* [47].

Various signaling pathways work together to maintain CSC properties. In the noncanonical Notch signaling pathway, NICD can be independent of CSL and interact with Wnt, Hippo or TGF-β Pathways to regulate the transcription of target genes [60]. The findings show that β-Catenin activity depends on YAP signal transduction and controls CSC program in basal breast cancer, which reflects the connection between Wnt pathway and Hippo pathway [77]. During the EMT program induced by TGF-β signal, Notch, Shh, Hippo and Wnt pathways were observed to be activated and linked, and Notch signal served as a key driver [78].

TFs interact with signaling pathways flexibly. It is shown that the activation of Wnt/β-Catenin pathway can up-regulate the expression of *OCT4*/*NANOG* in CSCs of NSCLC, which induce multi-drug resistance (MDR) and EMT [79]. The combination of SOX2 and β-Catenin improves the nuclear expression and transcriptional activity of *β-Catenin*, which is conducive to mediating the characteristics of CSCs and chemical resistance in CRC [39]. FOXM1 can enhance the stability of β-Catenin to activate Wnt pathway, thus affecting the stemness of GC [52]. An experiment suggest that Notch signal can induce *SOX9* expression to promote *SOX2* transcription, and *SOX2* up-regulation reduces the methylation level of *NOTCH1* promoter, which form a positive feedback loop to control the invasion of CSCs into the white matter tract in GBM [80]. As mentioned above, downstream TF of Hh pathway——GLI, can enhance the expression of *SOX2* and *NANOG* to regulate CSC stemness [63, 64].

It is remarkable that several other considerable signaling pathways participate in the transcription of CSCs and form a complex regulatory network. Numerous cytokines and growth factors transmit signals through JAK/STAT (Janus kinase/signal transducer and activator of transcription) pathway, including tyrosine kinase related receptors, tyrosine kinase JAK and TF STAT [81]. When JAK/STAT pathway is activated, STAT combines with cyclin promoter to enhance transcription, which can maintain CSC growth and reduce DNA damage in CRC [82]. Downstream effector molecules of JAK/STAT pathway include SOX2, NANOG and other stem TFs [83], STAT3 has been proved to promote up-regulation of Wnt ligand transcription [84]. Moreover, it is found that C-MYC/AMBRA1/STAT3 axis regulates cancer stemness and invasion potential in MB [48].

PI3K/AKT/mTOR pathway is rarely studied in CSCs, but it is reported to be cross linked to a variety of TFs and signaling pathways. The components of PI3K/AKT/mTOR pathway are phosphatidylinositol-3-kinase (PI3K), serine/threonine kinase (AKT, acts as key effector), mammalian target of rapamycin complex (mTORC, composed of mTOR, raptor, mLST8, PRAS40 and DEPTOR) [85]. The PI3K-AKT-mTOR cascade reaction is involved in regulating the activity of β-Catenin and several TFs (SOX4 and FOXO3) binding to β-Catenin [86]. The Wnt signal can interact with PI3K-AKT-mTOR pathway to co-regulate the expression of *MYC*, Hippo pathway also become a tie to link them [86]. In addition, it is reported that inhibiting AKT1/2 reduces *NANOG* expression in sarcoma CSC to reverse chemotherapy resistance [37], NANOG mediates the radio-resistance of tumor cells through AKT and Notch pathways partially [87].

NF-κB (nuclear factor-kappa B) pathway is a series of TFs composed of five protein members—p65, RelB, c-Rel, NF-κB1 and NF-κB2, which is related to inflammation and cancer [88]. NF-κB pathway directly regulates the transcription of *MYC* and promotes the stem phenotype of esophageal squamous cell carcinoma (ESCC) [89]. The phosphorylation of AKT activates NF-κB and enhances NF-κB nuclear translocation, affecting the progression of EMT and promoting ovarian cancer metastasis [90]. Research suggests that OCT4 may pass through AKT-NF-κB pathway to regulate angiogenesis in CSCs of HCC [32].

Peroxisome proliferator-activated receptor (PPAR) is a nuclear TF induced by ligands that plays a role in metabolism and cancer, including PPARα, PPARβ/δ and PPARγ [91]. PPARα is responsible for activating Wnt/β-Catenin signaling to initiate CSCs in HCC [92]. PPARδ is capable of binding to the *NANOG* promoter and enhancing the expression to amplify CSCs and promote CRC metastasis [93].

Fibroblast growth factor/FGF receptor (FGF/FGFR) signaling regulates the proliferation and differentiation of various types of cells, overexpressing and mutating in tumors [94]. FGF signaling is not dependent on any exogenous Wnt or TGF regulation and serves as the main regulator of self-renewal in CSCs [95]. The release of FGF2 induces zinc finger E-box binding to homologous box 1 (ZEB1) and SOX2 through FGFR1 signaling to maintain the CSC of GBM [96]. YAP1 up-regulates *FGFR1* expression at transcription level (in the promoter) via the TEAD binding site, while bFGF/FGFR1 induces *YAP1* expression through LATS1, revealing the connection between the FGF pathway and the Hippo pathway in CSCs [97]. An intriguing finding suggests that FGFR2 interacts with AKT to maintain the differentiation status of non-CSC cells in ESCC [98].

TFs and signaling pathways interfere with each other, jointly regulating CSC characteristics at the transcriptional level, and have been reported in various types of common cancers. Based on the dysregulation of different TFs and signaling pathways in various types of cancer, we have summarized the impacts and effects on CSC in extensively studied cancer types (Table 1).

**Table 1:** The dysregulated TFs or signaling pathway of CSCs in distinct cancers

Cancer types	Disregulated TFs or pathways in CSCs	The measures taken to target TFs or pathways	Effects generated on CSCs and tumors
Head and neck squamous cell carcinoma (HNSCC)	OCT4, NANOG, FOXM1	directly knock down *OCT4*; inhibit *DDX3* (human DEAD box RNA helicase) to achieve demethylation of m^6^A RNA in *FOXM1* and *NANOG*	decrease radiation induced DDR and inhibit radio-sensitivity of HNSCC cell lines [30]; impair the resistance to cisplatin [99]
Breast cancer	SOX2, Wnt/β-Catenin, Hippo	use small-molecule inhibitor of neddylation MLN4924 to down-regulate *SOX2*; knock down *YAP*	suppress the formation of tumor sphere and increase sensitivity to tamoxifen [35]; reduce the expression of many Wnt target genes and hinder the initiation and growth of CSCs [77]
Hepatocellular carcinoma (HCC)	NANOG, KLF4, Wnt/β-Catenin, PPARα	knock down *ABI2* to inhibit the transcription of *NANOG* and *KLF4*; use 4-phenylbutyric acid (4-PBA) to upregulate *PPARα* by activating β‐catenin	inhibit self-renewal and tumorigenicity of CSCs, growth and migration of HCC [38]; initiate CSCs to promote tumorigenesis [92]
Clear cell renal cell cancer	MYC	block estrogen receptor (ERβ) to target *C-MYC* mRNA through non-coding RNA	reduce CSC population and inhibit CSC phenotype [100]
Non-small cell lung adenocarcinoma (NSCLC)	OCT4, NANOG, Wnt/β-Catenin, STAT3, Hippo, FGF/FGFR	knock down *OCT4* or *NANOG* or *β-Catenin*; apply heat shock protein (HSP) 90 inhibitors to degrade STAT3 protein; knock down *YAP*	decrease *β-Catenin* expression and the target gene *CyclinD1*, make cells sensitive to anticancer drugs and eliminate EMT process [79]; inhibit STAT3 mediated activation of Wnt signaling, disrupt the vitality and migration of NSCLC cells [84]; up-regulate *FGFR1* transcription, inhibit self-renewal and eliminate CSC characteristics [97]
Glioblastoma (GBM)	SOX2, Notch, FGF/FGFR	knock down *SOX2* or *NOTCH*, knock down FGFR or apply FGFR antibodies	obstruct the distribution and invasive phenotype of CSCs [80]; inhibit the expression of *SOX2* and reduce tumorigenesis [96]
Prostate cancer	SOX2, NANOG, JAK/STAT, Notch, AKT	knock down *IFIT5* (as interferon induced gene and downstream effector of the JAK-STAT signaling pathway) to target SOX2 and NANOG; directly knock down *NOTCH1* or AKT	reduce the initiating ability of tumors and decrease prostasphere formation [83]; inhibit radiation resistance of tumor cells [87]
Esophageal squamous cell carcinoma (ESCC)	MYC, NF-κB, AKT, FGF/FGFR	activate NF-κB signaling with extracellular vesicles containing FMR1-AS1 to promote *C-MYC* expression; knock down *AKT* or *FGFR2*	mediate the transformation and migration of CSCs [89]; induce EMT and enrich CSC population [98]
Ovarian cancer	AKT, NF-κB, PPARα	use mono(2-ethylhexyl) phthalate (MEHP) to activate the PI3K/AKT/NF-κB pathway	promote cancer cell metastasis and induce EMT (inhibition of PPARα reverses this effect) [90]
Colorectal cancer (CRC)	SOX2, FOXM1, NANOG, PPARδ, FGF/FGFR	target RBM17 (a member of the spliceosome complex) to regulate selective splicing of *FOXM1* and promote *SOX2* transcription; use PPARδ agonists to induce *NANOG* expression; add FGFR inhibitors	enhance the stemness of CSCs and sphere formation [101]; induce CSC amplification and accelerate liver metastasis in vivo [93]; suppress self-renewal and long-term organoid [95]

### Targeting TFs or signaling pathways in CSCs

**Fig. 1:**
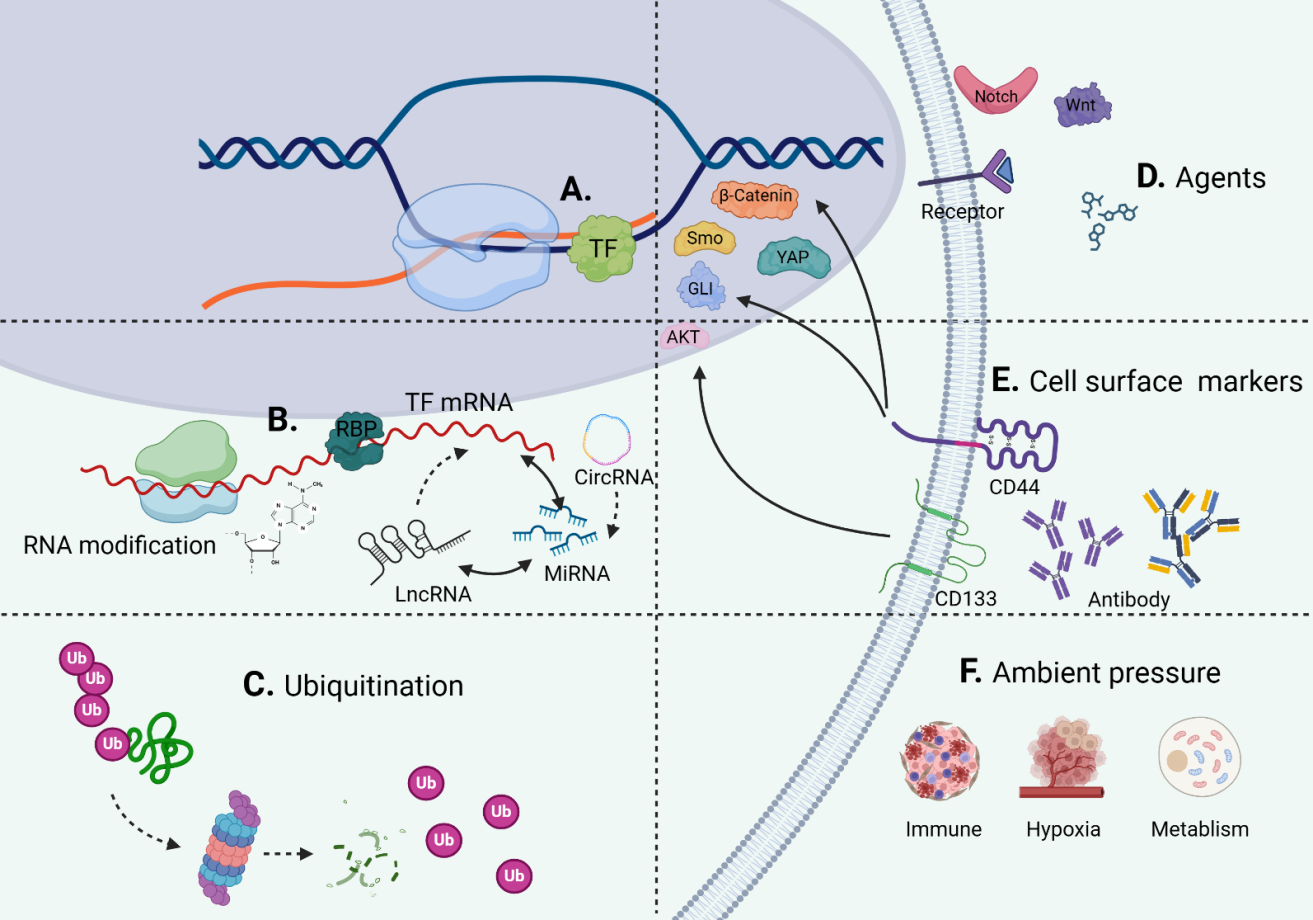
Targeting TFs in CSC from multiple perspectives **A **Directly blocking the combination of TFs and DNA: mainly through epigenetic mechanisms. **B** Targeting TFs mRNA to regulate the level: miRNAs target the inactivation of TF mRNA, and TFs mediate miRNA transcription. LncRNA influences TF mRNA through miRNA and forms ceRNA with miRNA. CircRNA targets TF mRNA through miRNA. RBP binds TF mRNA to affect the stability and translation. The modification of TF mRNA may play a role. **C** Promoting TFs degradation by ubiquitination. **D** Modulating various signaling pathways to affect TFs transcription and activity by diverse agents: it is effective for the TFs to regulate transcription of CSC by acting on ligands, receptors, complexes, and other TFs of various signaling pathways. **E** Inhibiting cell markers that regulate TFs gene expression. **F** Inducing ambient pressure such as metabolism, hypoxia and immune response to cause TFs inactivation

In order to target TFs to regulate the transcription of CSCs and obtain clinical benefits, there are several methods and strategies as follows: (1) Directly blocking the combination of TFs and DNA; (2) Targeting TFs mRNA to regulate the level; (3) promoting TFs degradation by post-translational modification (PTM); (4) effects on TFs transcription and activity by modulating various signaling pathways; (5) inhibition of cell markers that regulate TFs gene expression; (6) Inducing ambient pressure such as metabolism, hypoxia and immune response to cause TFs inactivation (Fig. 1). Next, the experiments and evidences of (2), (3), (4) will be listed, and the relevant contents of (1), (5), (6) will be demonstrated in other paragraphs.

### mRNA of TFs in CSCs as a target for regulation

There are various measures to target TFs mRNA in CSCs to modulate the level, thereby having an impact on the transcriptional regulation of CSCs, including non-coding RNA, RNA binding protein (RBP), and RNA modifications (Fig. 1B).

MicroRNAs (miRNAs) are a class of small non-coding RNA molecules that regulate gene expression by degrading mRNA through RNA induced silencing complex (RISC) formed by binding to 3’ UTR elements in mRNA [102]. MiRNAs can be used as tumor suppressor or tumor promoter, the correlation with miRNA and CSCs, EMT and drug-resistance has been widely studied [103]. It is reported that miRNA can directly target the inactivation of *NANOG* mRNA, or affect the related TF and pathways to indirectly regulate *NANOG* expression (such as Wnt/β-Catenin, Notch and JAK/STAT pathway) [104]. Moreover, the network of TFs and miRNAs cross-linking is gradually established. Research indicates that miR-145 exerts anti-stemness and tumor inhibitory effects by directly targeting *OCT4*, *NANOG*, *SOX2* and *KLF4*, conversely, these pluripotent TFs can also induce or inhibit the transcription of diverse miRNAs [27].

LncRNA is a long non-coding RNA with a length of more than 200 nucleotides. It is a key factor in gene regulation and also involved in the function of CSCs and EMT [105]. LncRNA OIP5-AS1 can directly interact with *OCT4* mRNA, enhance the stability of *OCT4* mRNA to increase *OCT4* expression, and endow lung cancer cells with CSC characteristics [106]. The interaction between lncRNA and miRNA forms a competitive endogenous RNA (ceRNA) mode and possesses a negative feedback regulation mechanism. LncRNA can target miRNA to regulate EMT and the expression of stem factors *NANOG*, *OCT4*, *SOX2* and *C-MYC*, as well as Wnt signal [107]. LINC00511 serves as the ceRNA of miR-185-3p to target E2F1 protein and bind it to the promoter region of *NANOG*, thus promoting transcription and enhancing the stemness of CSC in breast cancer [108].

Circular RNAs (circRNAs) are a major class of single stranded non-coding RNAs. Its occurrence depends on the canonical spliceosome mechanism to form circular conformation, which is stable in cells [109]. CircRNA is dysregulated in cancer progression and plays a role in cancer by acting as a sponge for miRNAs and interacting with RNA binding proteins [110]. Current studies discovered that circRNAs facilitate the characteristics of CSCs through miRNAs. The experiment proved that circFAM73A regulated miR-490-3p and recruited stem cell factor HMGA2 (high mobility A2 group, the downstream target of miR-490-3p) to promote the stability of β-catenin, thereby enhancing the stemness of CSCs and the drug-resistance in GC [111]. In addition, it is exposed that circ_ 001680 promotes the population of CSCs and drug-resistance in CRC by regulating miR-1 to target stem factor BMI-1 [112]. Last but not least, multiple studies indicated that gut microbiota regulates CSCs by targeting diverse TFs(SOX3/4/9, STAT3) through circRNA/miRNA network [113].

RBP can regulate the modification and translation of mRNA and affect the processing of miRNA and circRNA, playing a key role in cancer progression and metastasis [114]. Musashi-1 (MSI1), as a RBP and a CSC biomarker, can increase downstream RNA stability and interact with miRNA, thereby altering the expression and activity of OCT4 and STAT3 [115, 116]. Studies have shown that RBP La stimulates the post-transcriptional expression of TF ZEB1 and mediates TGF-β Induced EMT and stemness of CSCs [117]. Hu antigen R (HuR), as RBP, binds to snail mRNA to promote the stability of snail mRNA and the enhancement of snail protein expression, which encourages the formation of EMT and CSC in pancreatic cancer [118].

The modification of mRNA is recognized to have a direct impact on gene expression and is misregulated in cancer [119]. Among over 170 identified RNA modifications, inosine, 5-methylcytosine (m^5^C) and N6-methyladenosine (m^6^A) have been shown to coordinate the regulatory gene network within CSC to influence its fate [120]. However, there has not been any direct research on TF mRNA modification in CSCs, but rather a focus on fat mass and obesity related proteins (FTO). FTO exerts effect on the characteristics of CSCs in CRC and ovarian cancer as a demethylase targeting m^6^A modification of mRNA [121, 122], which provides inspiration for further research.

### The regulation of TFs in CSC through PTM

Ubiquitination is one of the important types of PTM and the main focus of current research on regulating the TFs of CSC through PTM (Fig. 1C). It is indicated that ubiquitination participates in mediating the transcriptional regulatory network with CSCs and EMT, E3 ubiquitin ligase can modulate the expression level of these pluripotent TFs by ubiquitination alone or ubiquitination combined degradation [123]. Due to the undruggable property of TFs, it is shown that targeting ubiquitin proteasome system (UPS) to inhibit the TFs expression is a potential alternative strategy [124]. For example, receptor for activated C kinase 1 (Rack1) directly binds to NANOG, thus preventing it from recruiting E3 ubiquitin ligase and ubiquitin dependent degradation to maintain CSC stemness [125].

WB100 targets C-MYC protein degradation through CHIP-mediated proteasome pathway and is currently conducting a phase I clinical study for the treatment of advanced solid tumors (NCT05100251).

### The use of signal transduction modulators to regulate the TFs and properties of CSCs

Multiple signal agents have been developed to block pathway conduction, which is applied to inhibit CSCs in distinct tumors (Fig. 1D). And some agents are being put into clinical trials and are expected to be combined with anticancer drugs for treatment in the near future.

Wnt signaling pathway inhibitors are widely used, mainly targeting wnt ligands and receptors, as well as β-Catenin [126]. Small molecule Wnt inhibitor ICG-001 can antagonize β-Catenin to inhibit CSCs in CRC and renal cell carcinoma [127, 128]. XNW7201 has been considered a potential new drug for advanced solid tumors due to the ability to block Wnt protein activity, and corresponding clinical trials have been conducted (NCT03901950). The inhibitors of the Notch signaling pathway in CSC research mainly target γ secretory enzymes or Notch ligands [126, 129]. The use of Notch inhibitor DAPT (γ secretory enzymes inhibitor) has been shown to reduce the number of renal CSCs and repress growth [128]. Moreover, it is reported that NADI-351 is an oral and effective Notch1 transcriptional complex inhibitor causing CSC ablation in mouse model [130]. Some Notch inhibitors have been applied in clinical trials to prevent cancer growth, such as CB-103 (NCT0574899), Nirogacestat (NCT0556798), AL101 (NCT04973683). The Hh pathway inhibitors applied to CSCs mainly target SMO and GLI [126, 131]. Experiments indicated that GANT61 as a GLI inhibitor significantly reduces the proportion and activity of CSCs in triple negative breast cancer (TNBC) [132]. A phase I clinical trial used docetaxel combined with SMO inhibitor (Sonidegib) to treat patients with metastatic TNBC. These 12 patients have previously received standard chemotherapy with taxanes and/or anthracyclines, of which 3 have achieved clinical benefits, and 1 patient has experienced a complete reponse [133]. Clinical trials of Sonidegib for the treatment of other advanced solid tumors are currently underway, such as NCT04007744 and NCT04066504. YAP/TAZ-TEAD acts as a tumor promoter in the Hippo signaling pathway, which is an important target for inhibiting the Hippo pathway in CSCs. YAP1 small molecule inhibitor Vitipofen has been proved to significantly block CSC properties [134]. The findings manifested that CA3 can represent a new inhibitor of YAP1 to significantly inhibit YAP1/TEAD transcriptional activity, targeting radiation-resistant esophageal adenocarcinoma cells with CSC characteristics [135]. It is suggested that statins inhibit the Hippo pathway by accelerating YAP phosphorylation and inducing inactivation in malignant mesothelioma (MM), which provides a potential choice for targeting CSC [136]. IAG933 can interfere with the interaction between YAP-TEAD proteins and entered clinical research in 2021 to treat advanced mesothelioma and other solid tumors (NCT04857372). In addition, a phase I clinical trial evaluating IK-930 (oral TEAD inhibitor) for the treatment of advanced solid tumor patients has been conducted (NCT05228015).

Besides, the joint use of signal transduction modulators displays significant effectiveness in hindering CSCs. For example, GANT61 combined with mTOR inhibition effectively reduces the viability of CSCs and the sphere formation of resistant cell lines in pancreatic cancer [137]. The mixture of Wnt pathway inhibitor PRI-724 and Hh pathway inhibitor vismodegib causes cell cycle arrest and downregulates the expression of *OCT4* in CSCs [138]. GSK-3β and MEK inhibitors can promote tumor growth and activate the expression of downstream Wnt signaling factors, and their combined use has been shown to affect CSC maintenance and radiation sensitivity in cervical cancer [139]. However, a clinical trial of RO4929097 (a Notch inhibitor) combined with Vismodegib in the treatment of advanced sarcoma showed that although the combination therapy was well tolerated, Vimodegib reduced the plasma concentration of RO492909 and did not significantly improve the progression free survival of patients [140].

### Epigenetic in the transcriptional regulation of CSCs

Epigenetics is reversible gene regulation method that involves variation in non-DNA sequences to affect transcriptional activity, the mechanisms include alterations in DNA methylation, histone modification, chromatin remodeling and non-coding RNA [9]. The generation of CSCs requires a reversible and heritable process to produce asymmetric division, which is of great significance for cancer initiation [141]. Epigenetic regulation of DNA accessibility and transcription is revealed to be involved in maintaining the characteristics of CSCs and promoting the occurrence and progression of cancer. Besides, multiple studies have reported that epigenetic mechanisms can hinder TFs binding to DNA, thereby regulating the transcription of CSC.

### DNA methylation in CSCs

DNA methylation is a chemical modification that silences genes by introducing methylated groups. It occurs in CpG dinucleotide and is mainly catalyzed by DNA methyltransferases (DNMTs, DNMT1, DNMT3A and DNMT3B) [142]. Methyl-CpG-binding domain proteins can recruit histone‐modifying and chromatin‐remodeling complexes to change chromatin structure or DNA conformation and stability, or affect gene expression by regulating the recruitment of DNA binding proteins (TFs) [141, 142]. DNA methylation becomes unstable in tumors and tends to increase or decrease specifically in some CpG islands [143].

At present, the research on transcriptional regulation through DNA methylation in CSCs mainly focuses on the use of DNMT to guide the expression of CSC markers and stemness factors (Table 2). DNMT1 generally maintains methylation pattern and inhibits transcription. Targeting DNMT1 to regulate methylation of the CSC marker brain-expressed X-linked protein 1 (BEX1) can mediate Wnt/β-Catenin signaling to promote CSC self-renewal in HCC [144]. Experiments proved that down-regulating DNMT1 to reduce the methylation level of CpG in *NANOG* promoter can enhance the CSC capability of tumor cells, which is confirmed as an indicator of the conversion of non-CSCs to CSCs [145]. Decitabine is a drug that can consume DNMT1 and has been applied in multiple clinical trials to evaluate safety and efficacy, such as NCT03019003, NCT0266418, and NCT03875287. We can conjecture that hypomethylation is of great value in CSCs. However, it is intriguing that OCT4 and NANOG prefer combination with methylated CpG, which is enlightening for the transcriptional regulation of CSCs [143].

**Table 2:** The DNA methylation disorder of CSCs in distinct cancers

Cancer types	The measures taken to target DNA methylation	Dysregulation in cancer	Effects generated on CSCs and tumors
GBM	knock down *SOX2*	increase the methylation level of the *NOTCH1* promoter	inhibit Notch signal transduction and hinder tumor invasion [80]
HCC	use DNMT1 inhibitor (zebularine); utilize miR-135a to target DNMT1	reduce the methylation of *BEX1* promoter; lower the methylation level of the *NANOG* promoter	induce the activation of Wnt/β-catenin signaling and promote the stemness of HCC [144]; enhance the CSC capability of tumor cells and regulate the transition from non-CSCs to CSCs [145]
CRC	use DNMT inhibitor (5-AzaDC)	down-regulate hypermethylation of *SFRP1*	inhibit the aggregation and stemness of CSC and hinder the invasion and distant metastasis of CRC [146]

### Histone modification in CSCs

Histone is the fundamental structural protein of chromatin and forms nucleosome together with packaged DNA. The amino acid residues in the terminal tail of histone and spherical core domain can be modified to regulate gene expression, including methylation, acetylation, phosphorylation and ubiquitination [141, 147]. Histone modification can not only induce local structural changes of chromatin to directly activate or inhibit transcription, but also bind effector proteins or chromatin remodeling complexes to indirectly drive genome response [147] (Table 3).

**Table 3:** The histone modification disorder of CSCs in distinct cancers

Cancer types	The measures taken to target histone modification	Dysregulation in cancer	Effects generated on CSCs and tumors
Pancreatic ductal adenocarcinoma (PDAC)	apply HDAC inhibitor (domatinostat)	block the expression and function of *FOXM1*	target CSC compartment and alter redox homeostasis of CSC, induce antitumor effect and make PDAC sensitive to chemotherapy drugs [54]
HCC	apply histone demethylase JMJD2D; remove HDAC11	enhance the expression of CSC markers (*EpCAM* and *SOX9*); facilitate histone acetylation of the *LKB1* promoter region	enhance Wnt and Notch signaling transduction, promote self-renewal capability of CSC [150]; hinder cancer stemness, HCC progression and sorafenib resistance [152]
Breast cancer	knock down or inhibit HDAC1 and HDAC3; combination of DNMT inhibitor (5-azacytidine) and HDAC inhibitor (butyrate)	results in HDAC7 downregulation and inhibit the transcription of oncogenes; target CSCs to reactivate the tumor suppressor genes	suppress the phenotype of CSCs [153]; block tumor occurrence and growth [154]
CRC	exhaust HotairM1 (lncRNA)	lead to H3K27 trimethylation at the *HOXA1* gene promoter site and epigenetic silencing	induce H3K27 acetylation at the *NANOG* gene enhancer site to up-regulate the expression, promote self-renewal of CSC and tumor spread [157]

Histone methylation directly mediates or inhibits the recruitment of histone binding proteins to promote or inhibit the transcription of different gene sites, resulting in local or global changes in cancer [148]. It is reported that histone 3 lysine 4 (H3K4) trimethylation can positively regulate transcription, its deletion on OCT4 and SOX2 motifs restricts the growth and activity of CSCs in GBM [149]. Histone lysine demethylase 4D (JMJD2D) reduced the level of histone 3 lysine 9 (H3K9) trimethylation on the promoter of *EpCAM* (epithelial cell adhesion molecule) and *SOX9* to enhance their expression, thus promoting recruitment and trans-activation of β-catenin and NICD1 [150].

Histone deacetylases (HDACs) are often overexpressed in cancer, and they have been used as a target in CSCs to regulate transcription [151]. The deletion of histone HDAC11 promotes histone acetylation in its promoter region to increase the transcription and expression of *LKB1*, thus activating AMPK signaling pathway and inhibiting glycolysis pathway, leading to the inactivation of signaling pathways and disorder of stemness in CSCs [152]. HDAC7 inhibits histone 3 lysine 27 (H3K27) acetylation of super enhancers (SEs) and TFs stemness related genes in breast cancer stem cells to enhance transcriptional activity [153]. A combination of DNMT and HDAC inhibitors was used in mouse models, significantly reducing CSC abundance and prolonging the survival time [154]. A clinical study used HDAC inhibitor Chidamide, Decitabine and immune checkpoint inhibitors in combination for the treatment of recurrent/refractory non-Hodgkin's lymphoma and advanced solid tumors (NCT05320640).

In addition, histone lactylation has been shown to directly stimulate chromatin to regulate transcription and participate in tumor metabolism [155]. It is uncovered that the increased lactylation of H3 histone effectively promotes the progression of HCC and induces the tumorigenicity of CSCs [156].

### Chromatin remodeling in CSCs

Chromatin remodeling refers to the alteration of chromatin packaging state and nucleosome structure and position in the process of gene expression, which is regulated by a variety of complexes and factors, and the mechanism is not completely clear. Switch/sucrose-nonfermented (SWI/SNF) chromatin remodeling complex (also known as BAF complex) exerts effect on human cancer, which can promote gene activation or inhibit TFs binding [158].

There are few studies on the regulation of CSC transcription by chromatin remodeling (Table 4). The research shows that carcinogenic MUC1-C protein directly activates the components of BAF complex up-regulated by TF E2F1, resulting in the increased expression of *NOTCH1* and *NANOG*, which facilitates the self-renewal of CSCs in prostate cancer [159]. There is evidence that Zinc finger and SCAN domain containing 4 (ZSCAN4) induce chromatin remodeling of the pluripotent gene promoter and maintain the phenotype of CSCs, but the specific relationship is not clear [160].

**Table 4:** The chromatin remodeling disorder of CSCs in distinct cancers

Cancer types	The measures taken to target chromatin remodeling	Dysregulation in cancer	Effects generated on CSCs and tumors
Prostate cancer	utilize oncogenic MUC1-C protein to activate BAF complex	induce *NANOG* expression and drive Notch signal	promote CSC stemness and tumor progression [159]
HNSCC	aided by ZSCAN4 induction to facilitate chromatin remodeling	lead to a functional histone 3 hyperacetylation at the promoters of *OCT3/4* and *NANOG*	maintain CSC phenotype and increase CSC frequency [160]
TNBC	utilize oncogenic MUC1-C protein to promote chromatin remodeling	induce the expression of *OCT4*, *SOX2*, *KLF4* and *MYC*	drive the state and stemness of CSC [161]

### Non-coding RNA in CSCs

The role of miRNA, lncRNA and circRNA in CSCs has been described in detail, but there is limited research on the regulation of CSC epigenetics. Here are pieces of evidences of non-coding RNA in CSC epigenetics. Experiments proved that miR-135a can act on DNMT1 to reduce DNA methylation of *NANOG* promoter, thereby enhancing CSC capacity [145]. LncRNA HotairM1 recruited several TFs into the promoter of its target gene *HOXA1*, resulting in histone H3K27 trimethylation and epigenetic silencing of *HOXA1*, and then induced H3K27 acetylation at *NANOG* gene enhancer site to up-regulate its expression [157]. The accumulation of NANOG further inhibits HOXA1, thus forming a mutual regulatory loop to maintain the development and self-renewal of CSCs [157]. In addition, it is reported in previous study that lncTCF7 recruits SWI/SNF complexes onto the promoter of *TCF7* to trigger *TCF7* expression and activate Wnt signaling subsequently, thereby enhancing the tumorigenic ability of liver CSCs [162].

### DNA elements of CSCs

The cis-acting elements involved in gene activation and transcriptional regulation in DNA are mainly promoters and enhancers. Promoter is a DNA sequence recognized, bound and transcribed by RNA polymerase, which has a highly flexible transcription initiation site (TSS) architecture [163]. Promoter DNA sequence and its binding sites to specific TFs regulate transcription and mediate gene expression variability [164]. Enhancers can activate or enhance gene transcription by recruiting TFs, cofactors and chromatin complexes to promoters [165]. Enhancer RNA (eRNA) transcribed from enhancers has been manifested to facilitate tumorigenesis [166]. Promoter and enhancer act as elements of hypomethylation region to regulate gene expression [142]. People gradually realize that the difference between enhancers and promoters is not obvious, which possess similar chromatin and sequence structure [163, 165]. Some promoters have enhancer activity, and active enhancers can combine with more distal TFs at their boundaries to drive local transcription initiation [163].

The transcriptional regulation of CSC promoter usually facilitates the expression of pluripotent genes through combination with TFs or epigenetic pathway, which is summarized in detail in the previous article. It is worth noting that in addition to binding to RNA, RBP also exhibits a preference for gene promoters and interacts with TFs to regulate transcription [167]. For example, YY1 (Yin Yang 1, RNA dependent TF) and RBM25 (RBP involved in splicing regulation) have interactions, where RBM25 first binds to the target gene promoter and then recruits YY1 into these promoters [167]. YY1 emerges as important regulator in the expression and activity of SOX2, OCT4, BMI-1, and NANOG to affect the stemness of CSCs [168]. Besides, YY1 interacts with cyclin-dependent kinase 9 (CDK9) to regulate transcriptional elongation in GSCs, thereby inducing interferon response and reconnecting the tumor microenvironment of GBM [169]. This brings new insights into the transcriptional regulation of CSC.

In the field of CSC enhancer research, in addition to TF binding and epigenetic modification, SEs have gradually entered the vision of researchers. The enhancer cluster formed by the series of large linear enhancers is called SEs, which has higher transcriptional activation ability and has been identified as the driving factor of carcinogenesis [170]. Research demonstrates that SEs may be used as targets for the treatment of HNSCC by eliminating CSCs. It has been proved that bromodomain containing protein 4 (BRD4) recruits NF-κB p65 to form SEs on cancer stem gene, and their destruction can effectively inhibit the invasion and metastasis of CSCs [171]. Knockout of TNBC specific gene ANLN driven by SEs inhibits the stemness of TNBC [172].

### Environment changes affect transcriptional regulation of CSCs

During the process of tumor occurrence and progression, CSCs face pressure from the surrounding environment, hypoxia and metabolism (Fig. 1F). These stimuli can regulate the proliferation, differentiation, and migration of CSCs at the transcriptional level, which supplies new course and insights for cancer treatment.

### TME regulates the transcription of CSC

**Fig. 2:**
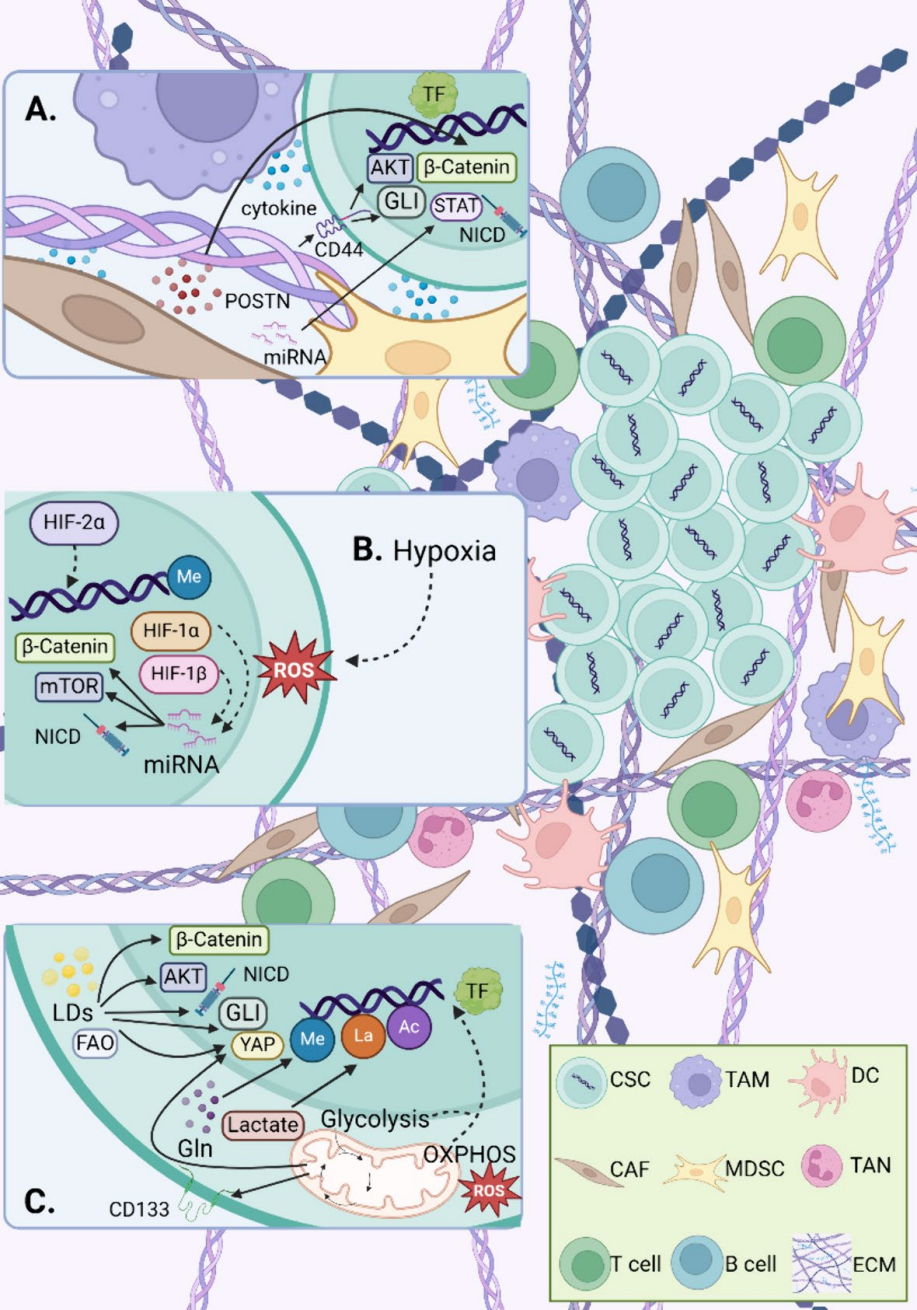
Multiple mechanisms of environmental stress regulating CSC transcription **A** The immune cells in TME activate the STAT, Wnt, AKT and Notch pathway of CSC by mainly releasing cytokines (IL-6) and inducing contact. CAFs secrete various substances (cytokines, POSTN, miRNA) to stimulate distinct pathways of CSC (Wnt/β-Catenin, PI3K/AKT/mTOR, STAT). ECM exerts effect on the PI3K and Hh pathways, as well as transcription of pluripotent TFs through mechanical stress and CSC markers (CD44). **B** Hypoxia causes the production of HIF and ROS. HIF-1 secretes miRNAs and activates the Wnt, Notch, PI3K/mTOR pathways, as well as acts on histone modifications. HIF-2 tends to directly regulate the gene expression of various TFs. Hypoxia induced ROS may support a transcription promoting role of CSCs. **C** OXPHOS occurring in the mitochondria of CSC can indirectly affect (through the release of ROS) or directly bear on the expression and effect of TFs. The alterations of mitochondrial function and state (mitochondrial fission and mitophagy) regulate the expression of TFs. The key enzymes in the CSC glycolysis process act on TF through the Hippo pathway and cell marker (CD133). Lactate metabolism plays a role in histone modification (lactylation) of CSC and ubiquitination of TF proteins. Lipid metabolism (LDs and FAO) is associated with the Wnt, Notch, Hippo, Hh and PI3K/AKT pathways and makes a difference in histone modifications (acetylation). Gln deprivation promotes pluripotent gene silencing and increases histone trimethylation

There are cancer cells surrounded by non-malignant cells, including a rich variety of immune cells, cancer associated fibroblasts (CAFs), vascular systems, extracellular matrix (ECM) and other types of cells that vary depending on the tissue in TME, which constitute a highly structured ecosystem [173]. These pert-cancer cells release growth factors, cytokines, chemokines, miRNAs, and extracellular vesicles to enhance the crosstalk between CSCs and TME, in order to achieve stemness and drug resistance [174]. CSC, differentiated cancer cells and various non-malignant cells interact to survive and invade in harsh ecological niches [9]. We will illustrate how diverse components of TME influence the transcriptional regulation of CSCs (Fig. 2).

### Immune cells regulate transcription of CSCs

CSC is proposed as a driving factor of intratumoral heterogeneity, and the negative associations between immune cell infiltration and intratumoral heterogeneity have been reported [175]. It is suggested that tumor associated macrophages (TAM), dendritic cells (DC), myeloid suppressor cells (MDSC), tumor associated neutrophils (TAN), lymphocytes, regulatory T cells, and cytotoxic T cells have an impact on the phenotype and function of CSCs [176]. Conversely, immune related signaling programs can be reprogrammed to promote tumor immune evasion through the communication between CSCs and immune cells [177]. In addition, CSCs exert effect on the processing and expression of antigens, secretion of humoral factors, upregulation of immunosuppressive molecules (immune checkpoints) and ligands and released particles of immune cells to create an immunosuppressive environment [178].

It is indicated that TAMs, MDSCs, and T cells mainly activate STAT proteins in CSCs by secreting various cytokines to regulate CSC stemness and induce CSC phenotype [179]. The secretion of IL-6 activates STAT3 to drive the production of CSC and EMT, which has received great attention [180]. Research shows that TAM derived IL-6 upregulates the expression of *SOX2*, *OCT4* and *NANOG* through STAT3 pathway, and induces CSC enrichment in breast cancer [181]. There is evidence that targeting the CSC marker DCLK1 inhibits IL-6/STAT3 signaling to hinder CSC traits and chemotherapy resistance in TNBC cells [182]. Besides, TAM derived IL-10 acts on JAK1, thereby activating STAT1/NF-κB/Notch1 signaling to promote CSC-like characteristics and tumor growth in NSCLC [183]. It is reported that MDSCs activate STAT3 signaling by stimulating the expression of colony stimulating factor 2 (CSF2) in epithelial ovarian cancer, thereby enhancing the transcription of *SOX2*, *NANOG*, *OCT4a*, *C-MYC*, and *KLF4*, as well as cancer stemness [184]. Napabucasin, as an effective STAT3 inhibitor, can downregulate the expression of CSC stemness genes and weaken the immunosuppressive ability of MDSCs, which has been reported to prolong the survival time of melanoma mice [185]. Napabucasin combined with traditional chemotherapy drugs in clinical trials has confirmed the safety and tolerance in the treatment of metastatic pancreatic cancer, metastatic CRC and unresectable HCC, but the efficacy is not obvious [186–188].

The transcription regulation of CSC by immune cells also involves other signaling pathways. For example, TAMs have been proved to promote the expression and activity of β-Catenin in TNBC cells to induce EMT and CSC characteristics, which is explained by activation of the AKT signal by TAM derived CCL2 (chemokine (C–C motif) ligand 2) [189]. An enlightening finding is that TAM induces stem phenotype by activating the Notch pathway after contact with non-CSC cells in living tumors, which plays a crucial role in the metastatic spread of CSCs [190].

### CAFs regulate transcription of CSCs

CAFs are activated fibroblasts with plasticity that can secrete regulatory factors to interact with immune cells and reshape the tumor ECM [191]. CAFs are divided into different subgroups with diverse surface markers. CD10 and GPR77 labeled CAFs subgroups can induce and maintain CSC stemness and chemoresistance in breast cancer and GC [192, 193]. Through paracrine signals and interactions with tumor cells, CAFs provide a support niche for CSCs [194] (Fig. 2A).

CAFs secrete periostin (POSTN) that activates protein tyrosine kinase 7 (PTK7) in HNSCC cells, leading to increased expression of β-Catenin and upregulation of CSC-like phenotype, proliferation and invasion [195]. The presence of CAFs can release cytokines to protect the PI3K/AKT pathway and transcriptional activation of CSCs in CRC [196]. CAF derived miR-146a-5p targets ARID3A and PRKAA1 in urothelial bladder cancer (UBC) cells to activate STAT3 and mTOR signals, promoting the stemness and chemical resistance of CSCs [197].

### ECM regulates transcription of CSCs

ECM is a non-cellular system composed of multiple macromolecules, providing mechanical clues and signaling molecules for tumors [198]. ECM stiffness is elaborately regulated by the environment and can act on various cells in TME to affect cancer progression [199]. It is reported that mechanical stress, environmental remodeling, regulatory factors and recruitment of non-malignant cells are involved in the regulation of CSCs by ECM [200].

There is evidence that breast cancer patients with stiff stroma have a lower response to drugs and a worse prognosis. It has been proved that matrix stiffness improves the transcriptional activity of *NANOG* through phase separation, leading to the enrichment of CSCs [76]. As one of the components of ECM, the binding of hyaluronic acid (HA) to CSC marker CD44 increases PI3K-4EBP1-SOX2 signaling, thereby enriching the CSC population and increasing CSC invasiveness [201]. An enzyme PEGPH20 that can decompose HA in combination with traditional chemotherapy for advanced pancreatic cancer is in clinical trial (NCT02921022). Tenascin-C (TNC) is a type of macromolecular ECM glycoprotein that has been shown to upregulate the CSC marker LSD1 and affect the characteristics of CSCs through the Hh signaling pathway, thereby promoting the migration and invasion in CRC [202].

### Hypoxia stress regulates the transcription of CSCs

CSCs and tumor cells are generally in a state of hypoxia during the occurrence and development of cancer. Hypoxia regulates the stemness and resistance of CSCs by mediating various TFs, signaling pathways, epigenetic modification factors and functional protein required for encoding CSC specifications [203] (Fig. 2B). Hypoxia inducible factors (HIFs) maintain oxygen homeostasis at the transcriptional level, which activates thousands of genes that mediate angiogenesis, CSC specifications, EMT, ECM remodeling and immune evasions [204]. HIF is characterized by oxygen reactivity α subunits (HIF-α) and stable β Subunit (HIF-β) composition, most studied in CSCs are HIF-1(α) and HIF-2α.

HIF-1α promotes angiogenesis, increase glycolysis reaction and affect apoptosis, which is mainly activated during acute hypoxia [205]. Multiple pieces of evidence prove that HIF-1 activates Wnt pathway through diverse means in CSCs. It is proved that stable knockdown of *HIF-1α* suppresses the Wnt pathway and reduces the expression of *NANOG* and *SOX2* in CSCs [206]. HIF-1ɑ regulated miR-1275 has been shown to activate Wnt/ β-Catenin pathway and Notch signaling thus promoting CSC phenotype and stemness in lung adenocarcinoma (LUAD) cells [207]. Besides, HIF-1 can directly activate the transcription of calreticulin (CALR) in cancer cells to induce Wnt/β-catenin signal transduction, which exert effect on promoting the phenotype of CSCs in breast cancer [208]. It is intriguing that HIF-1 activates transcription of pluripotent factor genes through epigenetic pathways. The increased expression of HIF-1 dependent S100A10 can form complexes and interact with histone demethylase KDM6A, ultimately being recruited to the OCT4 binding site to erase H3K27 trimethylation chromatin markers and promote transcription of *NANOG*, *SOX2* and *KLF4* in breast cancer [209]. This process can be induced by chemotherapy [209].

In addition, various other pathways have also been shown to be related to HIF-1. Research demonstrates that hypoxic conditions can reduce the tumor suppressor PTEN (phosphatase and tensin homologues), leading to activation of the PI3K/mTOR pathway and further accumulation of HIF-1α to obtain CSC and EMT phenotypes [210]. Hypoxia promotes the activation of the Shh pathway to promote the expression of *NANOG*, *OCT4* and *SOX2*, while HIF-1α silencing impairs the upregulation of Shh to inhibit the CSC population and EMT [211].

HIF-2α is more stable under chronic hypoxic conditions and tends to exhibit carcinogenic activity by regulating CSCs [205]. There is evidence that HIF-2α increases to the maximum value after 72 h of hypoxia, promoting expression of *KLF4*, *OCT4* and *β-Catenin* in CSCs [205]. MYC has been shown to regulate and bind to HIF-2, and NANOG and SOX2 may participate in this process to maintain self-renewal of CSCs [212]. Furthermore, chronic intermittent hypoxia exposure can activate mitochondrial reactive oxygen species (mtROS) and induce Bach1 (TF, BTB and CNC homology 1) to promote the expression of *NANOG*, *SOX2*, *OCT4* and cell markers in CSCs [213]. Currently, a variety of small molecule inhibitors targeting HIF-2α protein have been developed and also entered the clinical research stage, such as DFF332 (NCT04895748) and NKT2152 (NCT05119335).

In recent years, various drugs targeting the pluripotent TFs and signaling pathways of CSC have been developed, and it is proven that TME can synergistically inhibit the stemness of CSC. According to the stages of progress on these drugs, the table is summarized as follows (Table 5).

**Table 5:** Progress in drugs targeting CSC transcription——From cell experiments, animal models to clinical trials

Drug name	Target	Mechanism	Condition	Stage	Usage	Outcome
Ursolic acid	*C-MYC*	Increases *PTEN* expression to inactivate the FAK/PI3K/AKT/mTOR signaling pathway	Breast cancer	Experimental Study in vitro	Alone	Reduce the proportion of CSC and inhibit the migration and invasion of breast cancer [214]
Pongol methyl ether	*OCT4, NANOG*	Combine with AKT-1 to reduce signal transduction	Primary lung cancer	Experimental Study in vitro	Alone	Suppress spheroid formation and reduce CSC-specific marker expression [215]
Flavonoids (Nobiletin and Xanthohumol)	Wnt pathway	Reduce the activation of the Wnt pathway and down-regulate the expression of CD44	CRC	Experimental Study in vitro	In combination with 5-fluorouracil and oxaliplatin	Decrease stemness properties of CSCs and sensitize CSCs to standard chemotherapy [216]
MHY1485	mTOR pathway	Stimulate mTOR pathway and inhibit autophagy	Esophageal cancer	Experimental Study in vitro	Alone	Reduce CSC population and cancer stemness [217]
Atractylenolide I	PI3K/AKT/mTOR pathway	Inhibit the uptake of extracellular vesicles carrying miR-200c to influence signal pathway	CRC	Experimental Study in vitro	Alone	Obstruct the metastasis of CRC [218]
Thiamine tetrahydrofurfuryl disulfide	*SOX2, OCT4 and NANOG*	Inhibit the expression of stem gene	ESCC	Experimental Study in vitro and vivo	In combination with cisplatin/ concurrent chemoradiation therapy (CCRT)	Diminish stemness and enhance CCRT efficacy [219]
α-Mangostin	*SOX2, OCT4 and NANOG*	Induce mitochondrial apoptosis	Cervical carcinoma	Experimental Study in vitro and vivo	Monotherapy or in combination with Cisplatin	Synergically enhanced the cytotoxicity of cisplatin on CSC and reduce the stemness and proliferation of CSC [220]
A targeted co-delivery nanosystem of Salinomycin and doxorubicin	*OCT4, NANOG*	Redox-sensitive co-delivery micelles decorated with oligohyaluronic acid as the active targeting moiety down-regulate stem gene	Breast cancer	Experimental Study in vitro and vivo	Combination therapy	Reduce the risk of tumor metastasis and effectively alleviate splenomegaly caused by malignant tumors [221]
Sulforaphane	*SOX2, OCT4, Notch, SMO and GLI*	Inhibit the expression of CSC related stem gene	HNSCC	Experimental Study in vitro and vivo	In combination with Cisplatin or 5-fluorouracil	Inhibit CSC colony and tumor progression, enhance chemotherapeutics cytotoxicities against CSCs [222]
Rosiglitazone	*SOX9, SOX2, OCT4 and NANOG*	Up-regulate *ETS* homologous factor (EHF) to inhibit transcription	PDAC	Preclinical experiments in mice	Monotherapy	Inhibit cancer stemness, suppress the crosstalk between PDAC and CSCs, sensitise PDAC to gemcitabine therapy [223]
Glucosamine-labeled liposomal ceramide	*OCT4, SOX2, CD44 and CD133*	Target HIF-1α to block gene transcription	Lung cancer	Preclinical experiments in mice	In combination with paclitaxel and carboplatin	Three quarters of mice have a high tumor clearance rate [224]
Wortmannin	*SOX2, OCT4 and NANOG*	Inhibit PI3K/AKT/TBX3 signal transduction to down-regulate the expression of pluripotent gene	HCC	Preclinical experiments in mice	Monotherapy or in combination with Sorafenib	Reduce tumor size and weight [225]
Stattic	*OCT4*	Block STAT3 phosphorylation and activation, thereby reducing the expression of CCL16 to target the Wnt pathway	Breast cancer	Preclinical experiments in mice	Monotherapy	Inhibit tumor growth and decrease tumor volume [226]
PTC596	*BMI-1, OCT4*	Directly hinder the expression of *BMI-1*	Adenoid cystic carcinoma (ACC)	Preclinical experiments in mice	Monotherapy or in combination with Cisplatin	decrease the CSC fraction in tumors and prevent tumor relapse for 150 days [227]
SNS-032	*KLF4, C-MYC*	Inhibit cyclin dependent kinase 7/9 to obstruct transcription	Uveal melanoma	Preclinical experiments in mice	Monotherapy	Induce CSC elimination and reduce liver metastasis in tumor [228]
NHWD-870	*C-MYC, HIF-1α, TAM*	Inhibit BRD4 to target HIF-1α, hinder the expression of *C-MYC* and block the proliferation of TAM	Solid tumors and hematological malignancies	Preclinical experiments in mice	Monotherapy	Show strong anti-tumor activities in nine mouse models [229]
Emodin	TGF-β pathway	Block TGF-β signal transduction to inhibit pluripotent TF and act on TAM	Breast cancer	Preclinical experiments in mice	Monotherapy	Inhibit postoperative lung metastasis and significantly increase the survival [230]
INCB057643	*MYC*	Target BET to hinder the transcription of* MYC*	Advanced malignancies	Phase I/II clinical trial	Monotherapy or in combination with standard-of-care	Possess targeted toxicity and limited anti-tumor activity [231]
BMS-986158	*MYC*	Target BET to hinder the transcription of *MYC*	Advanced malignancies	Phase I/IIa clinical trial	Monotherapy	Yield tolerable safety and preliminary antitumor activity [232]
Nirogacestat	Notch pathway	Inhibit γ secretory enzymes	Desmoid Tumor	Phase III clinical trial	Monotherapy	Show significant benefits in various aspects [233]
CB-103	Notch pathway	Selectively inhibits the CSL-NICD interaction	ACC and hematologic malignancies	Phase I clinical trial	Monotherapy	Possess controllable safety and biological activity, but limited clinical anti-tumor activity [234]
Sonidegib	Hh pathway	Combine and inhibit SMO	Basal cell carcinoma	Phase II clinical trial	Monotherapy	Demonstrate sustained efficacy and a manageable safety profile [235]
Sonidegib	Hh pathway	Combine and inhibit SMO	Myeloid neoplasms	Phase I clinical trial	In combination with azacitidine	Confirm the safety of the combination but yield limited response rate in patients [236]
Napabucasin	*STAT3*	Directly inhibit gene transcription driven by STAT3	Metastatic colorectal cancer	Phase I/II clinical trial	In combination with pembrolizumab	Indicate antitumor activity with acceptable toxicities [237]
Decitabine	DNMT	Inhibit DNMT to lead to DNA hypomethylation and alter gene expression	Acute myeloid leukaemia	Phase II clinical trial	In combination with venetoclax	Show a manageable safety profile and high activity, need future larger and randomised studies [238]
Chidamide	HDAC, *Notch1, MYC*	Inhibit HDAC and down-regulate the level of the intracellular form of Notch1 and MYC	Angioimmunoblastic T-cell lymphoma	Phase II clinical trial	In combination with prednisone, etoposide, and thalidomide	Display effectiveness, tolerability, and economy [239]
Chidamide	HDAC, *Notch1, MYC*	Inhibit HDAC and down-regulate the level of the intracellular form of Notch1 and MYC	Advanced, hormone receptor-positive breast cancer	Phase III clinical trial	In combination with exemestane	Improve progression-free survival compared with placebo plus exemestane [240]

### Metabolic stimulation regulates the transcription of CSCs

CSCs control metabolic status through diverse transcription programs, and metabolic alternations can impact on the transcription of CSCs (Fig. 2C). A number of CSCs prioritize mitochondrial oxidative metabolism to complete biological activities [241]. A 2D in vitro system cultured cells with galactose based on forced OXPHOS to enrich pancreatic ductal adenocarcinoma CSCs, increase *NANOG* promoter activity and promote the expression of multiple pluripotent genes [242]. CSCs flexibly switch between oxidative phosphorylation (OXPHOS) and glycolysis as the main energy source to thrive in different settings [9, 243]. It is indicated that mitochondrial oxidative metabolism contributes to retention of the stems characteristics of CSCs, significantly inhibiting tumor spheroid formation in vitro and the expression of *NANOG*, *SOX2*, and *KLF4* by knocking down the key regulatory factor *PGC-1α* of mitochondrial function in cholangiocarcinoma [244]. Suppression of mitochondrial biogenesis by mitochondrial fission factor (MFF) has been proved to inhibit the activity of CSCs in breast cancer [245]. There is evidence that mitophagy is highly active in CSCs and tends to drive Notch1 signaling to promote CSC amplification [246]. It is shown that mitophagy promotes MSX2 (muscle segment homobox 2) turnover and mediates the stemness of oral cancer CSC through SOX2 [247]. Besides, the pharmacological inhibition of mitophagy leads to the accumulation and translocation of mitochondrial phosphorylated p53 to the nucleus, which binds to the *NANOG* promoter to inhibit expression and induces elimination of CSCs [248]. In addition, it is reported that OXPHOS derived ROS activates AKT to promote Wnt/β-catenin signaling in CSCs of CRC, which can be triggered by non-CSC derived lactate [249]. A clinical trial using OXPHOS pathway inhibitor ME-344 combined with bevacizumab for the treatment of recurrent metastatic CRC (NCT05824559).

In the case of sufficient oxygen, CSCs can mainly rely on glycolysis to obtain energy [243]. 6-phosphofructose-2-kinase (PFKFB3) serves as a rate limiting enzyme for glycolysis, the use of PFKFB3 inhibitors restrained the expression of *SOX2*, downregulated YAP/TAZ signaling, and enhanced the chemotherapy response of CSCs and EMT in SCLC [71]. Hexokinase 2 (HK2) is a key glycolytic enzyme that can interact with CD133 and promote the binding of ubiquitinase, thereby enhancing the stability of CD133 and boosting the expression of *OCT4* [250]. Lactate is a metabolic product of glycolysis, which has gradually been proven to involve transcriptional regulation of CSCs. Lactate level is capable of exerting effect on histone lactylation to regulate gene transcription, migration and apoptosis of CSCs [156]. The activation of lactate dehydrogenase A (LDHA) promotes the production of lactate and reduces the pH value, guiding the ubiquitination and stabilization of MYC mediated by the ubiquitinase to activate the *SLUG* promoter, which strengthens the characteristics of CSCs in breast cancer [47]. A purposeful discovery suggests that lactate coordinates PGC-1α to maintain OXPHOS activity and facilitate the transfer potential of normoxic CSCs in CRC [251].

Moreover, abnormal lipid metabolism is related to the function of CSCs [252]. A growing body of research shows that lipid metabolism plays a significant role in transcriptional regulation of CSCs. Damaged lipid droplets (LDs) biosynthesis has been shown to inhibit the expression of CSC markers and the EMT process [253]. There is evidence that accumulation of LDs activates PPARα in CSCs to sustain cancer stemness [254]. The enhanced adipogenic effect of human lung CSCs promotes the expression of TF *SPDEF* to drive *OPA1* transcription, which furtherance mitochondrial fusion and expression of *OCT4* in tumor spheres [255]. It is proved that lncROPM mediates lipid metabolism to activate PI3K/AKT, Wnt/β-Catenin and Hippo signaling to maintain CSC properties in breast cancer [256]. Stearoyl CoA Desaturase 1 (SCD1) is the central regulator of fat metabolism. The inhibitors of SCD1 downregulate the Wnt, Notch, and Hh signaling pathways and induce CSC death [257]. It is suggested that high expression of SCD1 enriches CSCs and increases the stemness and drug resistance in melanoma [258]. Under nutrient deficient conditions, CSCs can produce ATP through fatty acid oxidation (FAO) [252]. It is indicated that disturbance of FAO can target leukemia stem cells (LSCs) to affect their activity [259]. The FAO/ACLY (ATP citrate lyase) pathway has been reported to accelerate H3K27 acetylation of the *NANOG* promoter to induce dormancy of CRC cells, which provides new insights for further research in CSCs [260].

Glutamine (Gln) has been identified to be involved in regulating the fate of CSCs, with glutaminase (GLS) making a difference as a key target [261]. Targeting GLS1 and L-asparaginase to achieve Gln deprivation increases ROS levels and inhibits the Wnt/β-Catenin pathway, which impairs the tumorigenicity of CSC in vivo [262, 263]. It is shown that Gln deprivation leads to interactions between the expression of *MYC* members (*MYCN* and *C-MYC*), thereby damaging the phenotype and radiation resistance of CSCs in neuroblastoma [264].In addition, there is evidence that Gln deprivation through regulation α-Ketoglutaric acid (α-KG, a cofactor of chromatin modifying enzymes) contributes to increased H3K27 trimethylation to induce CSC gene dysregulation, indicating that Gln can affect the characteristics of CSCs through epigenetic pathways. Telaglynastat inhibit tumor cell growth in clinical studies as a GLS inhibitor, such as NCT03831932 and NCT03528642.

### Other factors regulating CSC transcription

**Fig. 3:**
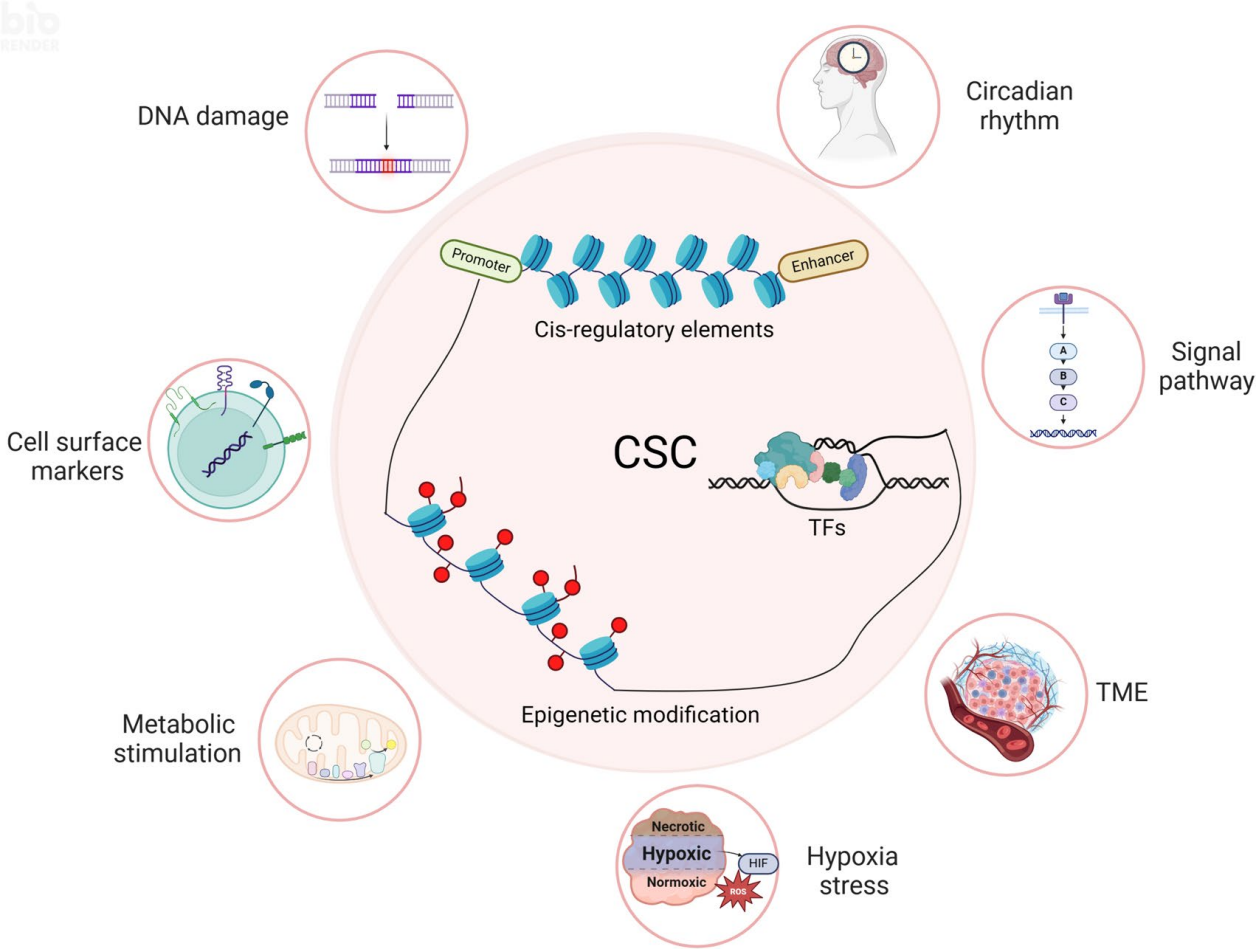
Regulating various aspects of CSC transcription from distinct perspectives The transcription of CSC involves TFs, epigenetic modification and cis-regulatory elements. Signal pathways, TME, hypoxia stress, metabolic stimulation, cell surface markers, DNA damage and circadian rhythm affect at least one of the three links to alter the phenotype and behavior of CSC

Based on recent relative research work, we feverishly discover that in addition to the participants in the transcription process and the influence of environmental stress, other factors involved in the regulation of CSC transcription alter the properties and biological behavior of CSCs, including cell markers, DDR and circadian rhythm (Fig. 3). Cell markers have long been identified as important targets for CSCs, and accumulating studies demonstrated their potential in transcriptional regulation. Effective DDR pathways protect CSCs from radiation and drug damage, while also playing a part in persistence of the stemness. The pleasant surprise is that the activation of circadian rhythm gene expression seems to be associated with the transcription of CSCs.

### The cell markers of CSC participate in transcriptional regulation

At present, there are no universal biomarkers for recognizing and identifying CSCs, but surface markers CD133, CD34, CD44, CD166, EpCAM and CD24 expression, as well as aldehyde dehydrogenase (ALDH) activity, have been widely used to isolate CSCs of distinct tumor types [176, 243]. However, it is necessary to strictly define the CSC state through self-renewal and functional characteristics of tumor initiation, rather than just marker expression. Various cell markers regulate transcriptional regulation to induce CSC stemness (Fig. 1E).

A previous study showed that CD133 deficiency disrupts the PI3K/Akt and Wnt signaling pathways and significantly reduces cell proliferation in ESCs [265]. CD133-AKT-Wnt/β-Catenin axis has been demonstrated to promote brain tumor-initiating cells in GBM [266]. It is reported that silencing of CD133 reduces the expression of the CSC stem gene *KLF4* in CRC [267]. Targeting CD133 can inhibit CRC resistance through the AKT/NF-κB pathway [268]. CD44 can activate the PI3K/AKT and Wnt pathways as well as *C-MYC* transcription to promote EMT and stemness, also interact with various ligands in TME to regulate the expression of *OCT4*, *NANOG* and *SOX2* [201, 269]. Studies have shown that CD166 can stimulate the activation of AKT and YAP signals, and knocking down CD166 in ovarian CSCs significantly reduces the expression of *OCT4* and *SOX2* [270]. In addition, other biomarkers not used for identification also target the signaling pathway of CSCs and the regulation of TFs. For example, CD73 can promote mRNA expression of stem related genes such as *NANOG*, *SOX2*, *OCT4*, *SOX9* and *C-MYC* in CSCs of HCC. The expression of *SOX9* is particularly affected, which is achieved by activating the AKT signaling [271]. According to reports, CD70 connects CD27 to LSC to induce activation of the Wnt pathway and trigger NF- κ B signal [272]. In a phase I clinical trial, targeting CD70 with cusatuzumab resulted in the elimination of LSC in patients with acute myeloid leukemia (AML) [273]. These 12 elderly AML patients who had not been treated in the past showed a significant reduction in LSC by up to 50 times after receiving cusatuzumab monotherapy. Then, these patients received treatment with cusatuzumab combined with azacitidine, with an overall response rate of 100% (8 cases achieved complete response). Currently, various antibodies targeting the surface specific marker CD123 of CSC are in clinical trials, including vibecotamab (NCT05285813), Flotetuzumab (NCT04158739) and SL-401 (NCT03113643). In addition, clinical studies combining EpCAM antibodies with chimeric antigen receptor T cell (CAR-T) technology are also underway, such as NCT02915445 and NCT05028933.

### DNA damage of CSC participates in transcriptional regulation

After immune and therapeutic mediated DNA damage, DNA repair reactions and DNA checkpoints are activated to enhance the resistance of CSCs [274]. Interference with DDR through Spironolactone has been proven to enable CSC to bypass resistance and result in growth inhibition [275]. It is reported that radiotherapy or chemotherapy induced DNA repair leads to tumor cells reprogramming into CSCs, which hastens the biogenesis of CSCs in GC [276]. DNA repair proteins can not only bind to target genes or TFs (such as KLF4 and MYC) to affect transcription programs, but also crosstalk with PI3K/AKT, TGF-β, Wnt and Notch signaling pathway to induce EMT and promote CSC evolution [277]. AZD6738 can inhibit Rad3 related proteins to target DDR and has been used in phase I/II clinical trials to treat solid tumor patients, including NCT05514132, NCT03682289, and NCT03579316. DNA double-strand break (DSB) is a crucial link in DNA repair response. The medium of homologous recombination repair (HRR), TONSL, exerts effect on DSB. Experiments demonstrated high expression of *TONSL* prompted co-amplification of *MYC* and upregulation of the transcription, which significantly influenced the survival of CSCs [278]. DNA damage checkpoint kinase (CHK) is gradually regarded as a target for CSCs. Research shows C-MYC targets CHK1 and CHK2 to inhibit DDR and eliminate CSC characteristics [279]. It has been proven that suppression of CHK1 sensitizes drug-resistant CSCs in CRC [280]. However, we still lack specific evidence that DNA damage checkpoints regulate CSC gene expression and induce CSC stemness.

### The core clock TFs of CSC participate in e transcriptional regulation

The circadian rhythm controls complex biological activities, and core clock genes play a crucial role in regulating the circadian rhythm. As main core clock TFs, BMAL1 and CLOCK monitor the cell cycle and metabolic homeostasis of CSCs in GBM [281]. Knockdown of *CLOCK* significantly reduces the expression of *OCT4* and *NANOG* and results in Wnt/β-Catenin pathway inactivation in CSCs of lung cancer [282]. According to the analysis, H3K27 acetylation and trimethylation modifications may mediate the binding of BMAL1 to the promoter, which enables reprogramming and drives new transcription functions [281]. It is proved that CLOCK and BMAL1 promote the infiltration of immunosuppressive microglia into TME through upregulation of chemokine *OLFML3* (CLOCK-directed olfactomedin-like 3) transcription to induce CSC stemness and support GBM [283]. Further research shows that this process may be achieved through CLOCK-OLFML3-HIF(1α)-LGMN(legumain)-CD162 axis [284]. Besides, experiments suggest that CLOCK-OLFML3-HIF-1α mediates upregulation of *POSTN* transcription, acting on TANK binding kinase 1 (TBK1) signaling in endothelial cells to induce angiogenesis [285]. However, there is evidence that BMAL1 inhibits the Hippo pathway but promotes Wnt signaling in intestinal CSCs, and the absence of BMAL1 activates YAP1 protein to enhance self-renewal [286].

## Conclusions and perspectives

In summary, CSC is a special cell group that can promote cancer progression and recurrence, assisting cancer cells continue to grow and proliferate under interference from chemotherapy and radiation therapy. In addition to excessive activation of DDR and alterations in cellular metabolism, the increased expression of ATP binding cassette (ABC) transporters in CSC enables drug efflux to avoid damage, thereby achieving MDR to hinder effective cancer treatment. The characteristic pluripotent TFs or signaling pathways in CSC interfere with each other, and the disorders lead to poor clinical prognosis in cancer patients, including OCT4, SOX2, NANOG, MYC, Wnt/β-Catenin, Notch, Hh, hippo, NF-κB and PI3K/AKT. Epigenetic modifications, particularly DNA methylation and histone acetylation, have been shown to exert effect on the transcription of pluripotent genes. The importance and effects of SEs are attracting people's attention. Other regulatory factors also play a significant role in TFs binding or signal transduction and the function of CSC, including non-coding RNA, RNA modification, TAM, MDSC, CAF, ECM, hypoxia, metabolism, cell markers, DDR, and core clock TFs.

A series of clinical trials targeting CSC transcription have been completed or are currently underway, covering agents targeting CSC surface biomarkers, TF protein targeting drugs, signaling pathway inhibitors and epigenetic modification enzyme inhibitors. In addition, some agents specifically for the microenvironment and metabolism have also been developed, which can affect the transcription and properties of CSC in a non-specific manner. Simultaneously targeting different links of CSC transcription can better inhibit cancer stemness and eliminate CSCs, such as the combination of signaling pathway inhibitors, or DNMT and HDAC inhibitors. Clinical administration tends to combine CSC transcription related agents with existing treatment regimens (chemotherapy, radiotherapy, targeted drugs, immune checkpoint inhibitors) to treat recurrent or refractory cancer patients. the application of non-coding RNAs, the epigenetic regulation and the promotion of environmental alterations. This illustrates that CSC serves as the key to cancer treatment and drug-resistance, and the transcription of CSC is the crux to targeting CSC.

Although targeting TFs and signaling pathways is currently vital and widely studied, regulating the transcription of CSC to improve cancer treatment efficacy faces many challenges. Firstly, TFs possess “undruggable” property and drugs that directly target the activity of pluripotent TFs in CSC have not yet been rigorously clinically validated. Secondly, based on the similarity of signaling pathways between CSCs and normal cells, not all signaling pathway inhibitors can be used in cancer treatment. Thirdly, the CSC model may not be applicable to all types of cancer and there are no unified and specific biomarkers for identification. Fourthly, the transcriptional regulatory network of CSC exhibits heterogeneity in distinct tumors, a large amount of research and evidence are required to investigate. Fifthly, non-coding RNAs play a great role in CSC transcriptional regulation, but available drugs have not yet been developed. Sixth, the microenvironment and metabolic characteristics of CSC are significant for regulating transcription, but such studies are mostly limited to non-specific cytokines and metabolic pathways. Seventh, there are already quite a few drugs and trials targeting CSC surface biomarkers, most of which can be tolerated by patients but have less than ideal therapeutic effects. It is supposed to find more valid therapeutic agents or treatment plans.

## Data Availability

Not applicable.

## Abbreviations

CSC: Cancer stem cell
TF: Transcription factor
TME: Tumor microenvironment
EMT: Epithelial-mesenchymal transition
MET: Mesenchymal-epithelial transition
EMP: Epithelial mesenchymal plasticity
GBM: Glioblastoma
VEGF: Vascular endothelial growth factor
DDR: DNA damage response
ESC: Embryonic stem cell
OCT4: Octamer-binding transcription factor 4
SOX2: Sry-related HMG box 2
KLF4: Kruppel-like factor 4
HNSCC: Head and neck squamous cell carcinoma
GSC: Glioma stem cells
HCC: Hepatocellular carcinoma
NSCLC: Non-small cell lung adenocarcinoma
HOX: Homeobox
ABI2: Abelson interaction factor 2
CRC: Colorectal cancer
MIZ-1: MYC-Interacting Zinc Finger Protein 1
MB: Medulloblastoma
FOX: Forkhead box protein
GC: Gastric cancer
Hh: Hedgehog
GPCR: Atypical G protein coupled receptor
LRP: LDL receptor related protein
DVL: Dishevelled protein
ROR: Receptor tyrosine kinase-like orphan receptor
NICD: Notch intracellular domain
CSL: CBF-1/suppressor of hairless/Lag1
DLL: Delta-like ligand
JAG: Jagged
PTCH: Patched protein
SMO: Smoothened protein
GLI: Glioma-associated oncogene homolog
MST1/2: Serine threonine kinase3/4 (STK3/4)
LATS: Large tumor suppressor kinase
YAP: Yes-associated protein
TAZ: Trascriptional coactivator with PDZ-binding motif
TEAD: Transcriptional enhanced associate domain
TGF-β: Transforming growth factor β
MDR: Multi-drug resistance
JAK: Janus kinase
STAT: Signal transducer and activator of transcription
PI3K: Phosphatidylinositol-3-kinase
AKT: Serine/threonine kinase
mTOR: Mammalian target of rapamycin
NF-κB: Nuclear factor-kappa B
ESCC: Esophageal squamous cell carcinoma
PPAR: Peroxisome proliferator-activated receptor
FGF: Fibroblast growth factor
FGFR: FGF receptor
ZEB1: Zinc finger E-box binding to homologous box 1
HSP: Heat shock protein
RBP: RNA binding protein
miRNA: MicroRNA
lncRNA: Long non-coding RNA
ceRNA: Competitive endogenous RNA
circRNA: Circular RNA
HuR: Hu antigen R
m5C: 5-Methylcytosine
m6A: N6-methyladenosine
FTO: Fat mass and obesity related protein
UPS: Ubiquitin proteasome system
Rack1: Receptor for activated C kinase 1
TNBC: Triple negative breast cancer
DNMT: DNA methyltransferase
BEX1: Brain-expressed X-linked protein 1
H3K4: Histone 3 lysine 4
JMJD2D: Histone lysine demethylase 4D
H3K9: Histone 3 lysine 9
EpCAM: Epithelial cell adhesion molecule
HDAC: Histone deacetylase
H3K27: Histone 3 lysine 27
SE: Super enhancer
SWI/SNF: Switch/sucrose-nonfermented
BAF: BRG1/BRM associated factor
ZSCAN4: Zinc finger and SCAN domain containing 4
TSS: Transcription initiation site
eRNA: Enhancer RNA
YY1: Yin Yang 1
CDK9: Cyclin-dependent kinase 9
BRD4: Bromodomain containing protein 4
CAF: Cancer associated fibroblast
ECM: Extracellular matrix
TAM: Tumor associated macrophage
DC: Dendritic cell
MDSC: Myeloid suppressor cell
TAN: Tumor associated neutrophil
CSF2: Colony stimulating factor 2
CCL2: Chemokine (C–C motif) ligand 2
POSTN: Periostin
PTK7: Protein tyrosine kinase 7
HA: Hyaluronic acid
TNC: Tenascin-C
HIF: Hypoxia inducible factor
LUAD: Lung adenocarcinoma
CALR: Calreticulin
PTEN: Phosphatase and tensin homologues
ROS: Reactive oxygen species
Bach1: BTB and CNC homology 1
ACC: Adenoid cystic carcinoma
OXPHOS: Oxidative phosphorylation
MFF: Mitochondrial fission factor
PFKFB3: 6-Phosphofructose-2-kinase
HK2: Hexokinase 2
LDHA: Lactate dehydrogenase A
LD: Lipid droplet
SCD1: Stearoyl CoA Desaturase 1
FAO: Fatty acid oxidation
LSC: Leukemia stem cell
ACLY: ATP citrate lyase
Gln: Glutamine
GLS: Glutaminase
α-KG: α-Ketoglutaric acid
ALDH: Aldehyde dehydrogenase
AML: Acute myeloid leukemia
CAR-T: Chimeric antigen receptor T cell
DSB: DNA double-strand break
HRR: Homologous recombination repair
CHK: Checkpoint kinase
OLFML3: CLOCK-directed olfactomedin-like 3
LGMN: Legumain
TBK1: TANK binding kinase 1
ABC: ATP binding cassette
